# Using Hierarchical Clustering of Secreted Protein Families to Classify and Rank Candidate Effectors of Rust Fungi

**DOI:** 10.1371/journal.pone.0029847

**Published:** 2012-01-06

**Authors:** Diane G. O. Saunders, Joe Win, Liliana M. Cano, Les J. Szabo, Sophien Kamoun, Sylvain Raffaele

**Affiliations:** 1 The Sainsbury Laboratory, Norwich Research Park, Norwich, United Kingdom; 2 Cereal Disease Laboratory, Agricultural Research Service, U.S. Department of Agriculture, St. Paul, Minnesota, United States of America; University of California Riverside, United States of America

## Abstract

Rust fungi are obligate biotrophic pathogens that cause considerable damage on crop plants. *Puccinia graminis* f. sp. *tritici*, the causal agent of wheat stem rust, and *Melampsora larici-populina*, the poplar leaf rust pathogen, have strong deleterious impacts on wheat and poplar wood production, respectively. Filamentous pathogens such as rust fungi secrete molecules called disease effectors that act as modulators of host cell physiology and can suppress or trigger host immunity. Current knowledge on effectors from other filamentous plant pathogens can be exploited for the characterisation of effectors in the genome of recently sequenced rust fungi. We designed a comprehensive *in silico* analysis pipeline to identify the putative effector repertoire from the genome of two plant pathogenic rust fungi. The pipeline is based on the observation that known effector proteins from filamentous pathogens have at least one of the following properties: (i) contain a secretion signal, (ii) are encoded by *in planta* induced genes, (iii) have similarity to haustorial proteins, (iv) are small and cysteine rich, (v) contain a known effector motif or a nuclear localization signal, (vi) are encoded by genes with long intergenic regions, (vii) contain internal repeats, and (viii) do not contain PFAM domains, except those associated with pathogenicity. We used Markov clustering and hierarchical clustering to classify protein families of rust pathogens and rank them according to their likelihood of being effectors. Using this approach, we identified eight families of candidate effectors that we consider of high value for functional characterization. This study revealed a diverse set of candidate effectors, including families of haustorial expressed secreted proteins and small cysteine-rich proteins. This comprehensive classification of candidate effectors from these devastating rust pathogens is an initial step towards probing plant germplasm for novel resistance components.

## Introduction

Rust fungi are a diverse monophyletic group of obligate plant pathogens that infect numerous economically important cereal crops and constitute a serious threat to global food security [1]. Currently, wheat stem rust is of particular concern due to the emergence of a highly virulent race, Ug99, first detected in Uganda in 1998 and characterized in 1999 [2]. The Ug99 race and its variants are estimated to be virulent on over 90% of the wheat grown globally, presenting a substantial threat to wheat production [2]. Rust fungi also present a serious threat to the production of bioenergy and fundamental plant products derived from the poplar tree. Indeed, poplar plantations are particularly susceptible to widespread infestation by the leaf rust fungus, with the threat exacerbated by artificial cultivation methods such as dense planting and breeding for uniformity, which limits genetic diversity [3]. Although fungicides can be used to manage rust fungi, the costs are considerable and often outweigh the benefits, particularly for developing nations. Therefore, the integration of new resistance (*R*) genes through plant breeding programs remains the main sustainable solution to dealing with these notorious and destructive plant pathogens.

The plant R proteins form a sophisticated surveillance mechanism that recognizes pathogen molecules as signatures of invasion and activates immune responses to halt colonization in resistant cultivars. However, few R proteins have been characterized that are active against rust pathogens. For stem rust *R* (*Sr*) genes, the introduction of the *Sr2*-complex in high-yielding wheat cultivars in the 1970s led to the termination of many wheat breeding programs, limiting the search for new *Sr* genes [2]. The Ug99 stem rust race group has overcome most of the key wheat resistance genes and some of the alien resistance genes that were previously incorporated, such as *Sr31* from rye, *Sr38* from *Triticum ventricosum* and *Sr24* from *Agropyron ponticum* [4], [5], [6]. Therefore, the identification of new resistance genes against these fungi has become a priority in crop research.

During infection, rust fungi, like many other plant pathogens, secrete effector proteins from specialized feeding structures known as haustoria [7]. These structures form invaginations of the plant plasma membrane, allowing an intimate contact with the plant. Once secreted, effectors can act either in the extrahaustorial matrix, the extracellular space or within the host cell cytoplasm to promote colonization and pathogenicity [8]. In cases where effector proteins are recognized by corresponding host R proteins, they induce an apoptotic cell death known as the hypersensitive response (HR), and they are considered to have an “avirulence” activity (AVRs).

Only a handful of effectors have been identified from rust fungi. Understanding the defining features of filamentous plant pathogen effectors should assist in the identification of additional effectors from rust fungi, particularly from economically important species. Effectors from oomycete pathogens that act within the host cell often contain a conserved host-translocation motif, which is essential for transport into the host cytoplasm [9]. Some fungal effectors, including effectors of flax rust fungi, are thought to be translocated into the host cytoplasm [10], [11], [12]. However, to date no universal host-translocation motif has been identified in fungi. Effectors that remain in the extracellular interface between the pathogen and the plant, and some host-translocated effectors, are small cysteine rich proteins (SCRs). They contain intramolecular disulfide bridges that likely stabilize protein tertiary structure in the harsh environment such as the plant apoplast. For example, the *Cladosporium fulvum* SCR effector protein Avr2 inhibits proteases within the tomato apoplast to promote virulence on cultivars lacking the corresponding resistance gene Cf-2 [13]. Some filamentous pathogen effectors such as *Magnaporthe oryzae* Pwl effectors and many oomycete RXLR effectors are repeat-containing proteins (RCPs) [14], [15]. RCPs have been proposed to be involved in the virulence of *Legionella* [16], *Candida* [17], *Fusarium* [18] and *Phytophthora* [19]. Effector genes are known to occupy unstable regions of genomes such as repeat-rich regions and centromeres [20]. For instance, effector genes are located within repeat-rich regions of the genome in the blackleg fungus *Leptosphaeria maculans* [21].

Classical strategies for the identification of fungal effectors include map-based cloning, analysis of fungal secretomes during infection, identification of HR-inducing pathogen genes, mutagenesis and screening of expressed sequence tag (EST) libraries [22]. However, these methods are typically labor-intensive and can be problematic. The release of the genome sequences of several rust fungi provides the opportunity to develop a comprehensive high throughput computational method for cataloguing their effector repertoires. These effector proteins can then be used as molecular probes to understand the basic biology of the plant-pathogen interactions and identify new R proteins [23], [24].

To identify *R* genes by use of effector proteins as probes in resistant plant cultivars, the complement of effector proteins must first be characterized. In this study, we took a first step towards identifying effectors from important rust fungal pathogen species, by annotating and classifying the secretome of two species. Eight defining features of effectors were used to classify secreted protein families. Hierarchical clustering was employed to rank the list of candidate families revealing secreted protein families with the highest probability of being effectors. We also highlight eight candidate effector families that fulfill the most prominent features of known effectors and that are high priority candidates for follow-up experimental studies.

## Results and Discussion

### Defining the effector repertoire of two rust fungi

**Figure 1:**
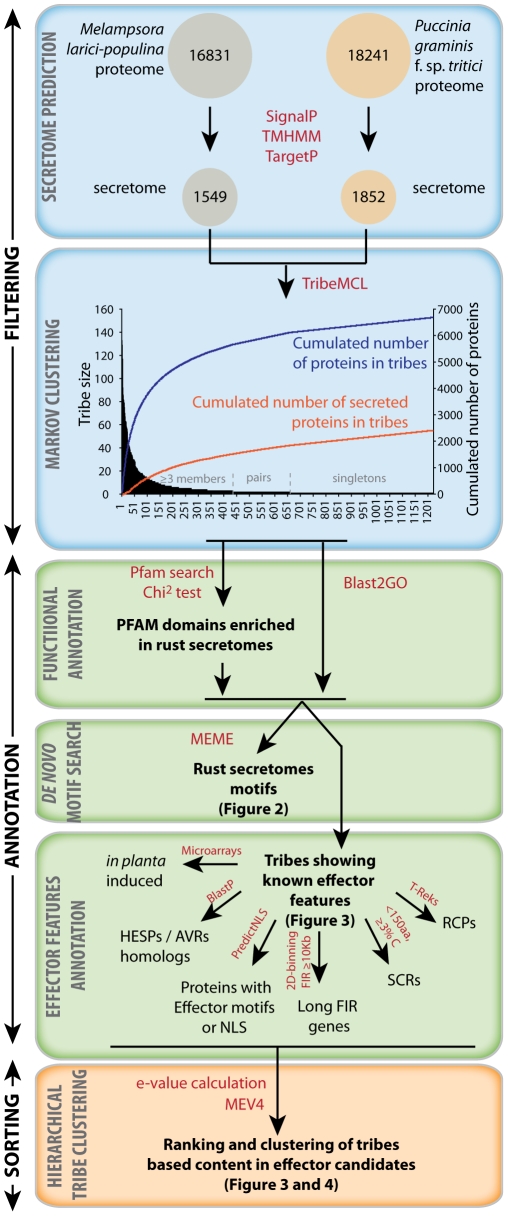
Bioinformatic pipeline for the clustering of secreted protein families and classification and ranking of effector candidates. The pipeline is composed of six major steps delimited by boxes. Step 1 (Secretome prediction) identifies secreted proteins from the predicted proteomes. A total of 1549 and 1852 secreted proteins were predicted from *M. larici-populina* and *P. graminis* f. sp. *tritici* proteomes, respectively. Step 2 (Markov clustering) groups secreted and non-secreted proteins according to sequence similarities to secreted proteins. A total of 435 secreted protein families (tribes) of at least 3 proteins were defined from the proteomes of the two rust fungi. Step 3 (Functional annotation) implements tribe annotations based on sequence homology searches. Step 4 (*De novo* motif search) searches for conserved motifs. Step 5 (Effector features annotation) uses the current knowledge of effector features to annotate individual members of secreted protein families. Step 6 (Hierarchical tribe clustering) ranks and classifies the tribes based on their content in proteins with effector features to provide a priority list for functional validation studies. Tools (programs and databases) used are indicated in red. HESP, haustoria expressed secreted protein; AVR, effector protein with defined avirulence activity; NLS, nuclear localization signal; FIR, flanking intergenic region; RCPs, repeat containing proteins; SCRs, small cysteine rich proteins.

To identify and classify candidate effectors of the poplar leaf rust fungus *M. larici-populina* and the wheat stem rust fungus *P. graminis* f. sp. *tritici*, we constructed a bioinformatic pipeline using current knowledge of the properties of validated filamentous plant pathogen effectors. Considering that effector proteins are often evolutionarily diverse and are rarely similar to characterized proteins [25], limiting the analysis to sequence similarity searches to known effectors is insufficient. Rather, we based the identification and classification of putative effector proteins on an array of features. The pipeline was organized into three major modules, (i) filtering, (ii) annotation and (iii) sorting (**Figure 1**). Using this pipeline we predicted 1549 secreted proteins from the proteome of *M. larici-populina* and 1852 for *P. graminis* f. sp. *tritici*. The two secretomes were combined and used as a template to group sequences, including non-secreted proteins from the two examined rust fungal species, into tribes using Markov clustering [26]. Similarity searches were undertaken using the mature protein sequences, to prevent unspecific clustering due to the N-terminal signal peptide region. A total of 1222 tribes containing at least one secreted protein were produced by analysis of the combined proteomes of *M. larici-populina* and *P. graminis* f. sp. *tritici*. Of these, 435 tribes contained at least three proteins. Pairs and singletons may contain effector candidates as some displayed similarity to *Melampsora lini* haustoria expressed secreted proteins (HESPs), such as Mellp_37347 (similarity to *M. lini* AvrL567) and Mellp_124274 (similarity to *M. lini* AvrP4) and were therefore retained for further analysis. A total of 6663 proteins were analyzed, of which 2826 contained an identifiable secretion signal. This work therefore significantly expands the analyses of 1405 small secreted protein tribes reported previously [27].

### *De novo* motif analysis reveals several conserved cysteine motifs in secreted proteins of rust fungi

**Figure 2:**
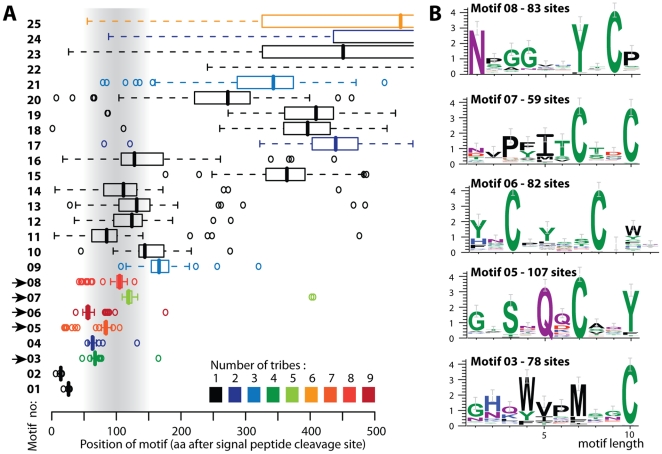
*De novo* motif searches in the secretome of *M. larici-populina* and *P. graminis* f. sp. *tritici* reveal conserved cysteine rich motifs. (A) Amino acid position of 25 motifs in the secretome tribes of rust fungi reported by MEME. Arrows highlight positionally constrained motifs that are abundant in the secreted protein tribes. Position is given after the signal peptide cleavage site when applicable. Grey shading indicates the expected position for putative host-translocation motifs (amino acids 50 to 150). Box plots show median position (bar) first and third quartiles (box), first values outside 1.5 the interquartile range (IQR) (whiskers) and outliers (dots), coloured according to the number of tribes containing at least two proteins harbouring the motif. Motifs are classified by decreasing IQR from top to bottom. (B) Sequence logos of motifs with the highest positional constraint, distribution over the largest number of tribes and greatest number of individual proteins (sites) containing the motif.

To detect conserved motifs in the secretome of *M. larici-populina* and *P. graminis* f. sp. *tritici* we used the program MEME [28]. We identified five positionally constrained motifs (motifs 03, 05, 06, 07 and 08) ranging in abundance from 59 to 107 sites (**Figure 2**). None of the motifs of the secretome of rust fungi reached the frequency of the *Phytophthora* RXLR or powdery mildew Y/F/WXC motifs (34.8% and 19%, respectively). The presence of one or two highly conserved cysteines was a common feature of the five motifs found.

Motif 03 contained W, M and C residues that were conserved and organized in a WXXMXXC pattern at median position 67 amino acids after the predicted cleavage site. This motif was identified in at least two proteins in each of four tribes (tribes 5, 227, 455 and 469) and in a total of 78 proteins. Motif 05 contained S, Q, C and Y residues in a SXQXCXXY pattern. Seven tribes (tribes 5, 158, 159, 208, 227, 366 and 455) contained at least 2 proteins with motif 05, which was identified in a total of 107 proteins at a median position of 84 amino acids after the cleavage site. This motif is also present in the largest tribe of small secreted protein of *M. larici-populina* reported in [27]. Motif 05 was the most abundant motif identified, found in 3.2% of the combined secretome of *M. larici-populina* and *P. graminis* f. sp. *tritici*. Motif 06 and motif 07 contained two conserved cysteines separated by 4 and 2 residues, respectively. Motif 06 was shared among the highest number of tribes (9, tribes 5, 76, 158, 209, 237, 362, 369, 455 and 469) and found in 82 proteins at a median position of 56 amino acids after the cleavage site. Motif 07 was shared between 5 tribes (tribes 5, 158, 227, 455 and 482) and found in 59 proteins at a median position of 119 amino acids after the cleavage site. Finally, motif 08 contained a conserved YXC pattern that was preceded by a conserved N residue. This motif was found in 8 tribes (tribes 5, 125, 158, 159, 227, 353, 366 and 562) and a total of 83 proteins at a median position of 105 amino acids after the cleavage site.

All motifs identified contained one or two conserved cysteines that may function in protein stability in the extracellular space [22], and several had conserved tyrosine residues, a feature reported in some host-translocated effectors [29]. Motifs 06 and 08 contained the Y/F/WXC sequence which has been proposed as a signature for a new class of effectors from haustoria-forming fungi [30] and has been reported as abundant in the secretome of *M. larici populina* and *P. graminis* f. sp. *tritici* [27]. These observations are consistent with the view that some effectors of rust fungi might be secreted into the apoplast first, where they would be processed and folded, before uptake by the plant cell [22], [31]. However, in spite of systematic unbiased search efforts such as reported here, clear translocation motif candidates are still lacking for effectors from haustoria-forming fungi. It is therefore tempting to speculate that a specific protein fold, matured in the extracellular space through the conserved cysteine motifs, might trigger uptake by the plant cell. As a consequence, conserved motifs seem of little help to identify effectors of rust fungi, prompting us to consider additional features of known filamentous plant pathogen effectors.

### Lineage-specific orphan protein families are abundant in the secretome of rust fungi

**Figure 3:**
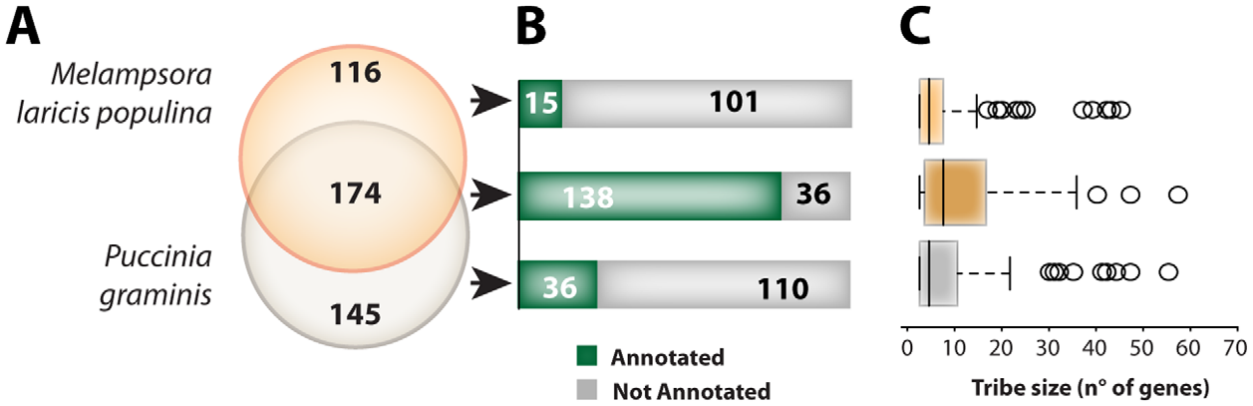
Comparison between core and lineage-specific secretome tribes of at least three proteins in rust fungi. (A) Core tribes (containing proteins from both species of rust fungi) represent forty percent (174 out of 435) of the secretome tribes containing three or more proteins. (B) Core secretome tribes of at least three proteins are enriched in proteins annotated by homology searches whereas lineage-specific tribes often remained non-annotated. (C) Size distribution of core secretome tribes of at least three proteins was shifted towards larger tribes compared to lineage-specific tribes. The same conventions as in Figure 2 were used in the boxplots.

The genomes of rust fungi contain numerous lineage-specific expanded protein families [27]. To investigate the distribution of shared and unique secreted proteins in the secretome of *M. larici-populina* and *P. graminis* f. sp. *tritici*, we investigated the species composition of the 435 tribes with three or more proteins in relation to annotation and number of proteins per tribe. Forty percent of the tribes with three or more members (174 tribes) contained proteins from both species (**Figure 3A**). These tribes constitute 941 secreted proteins that likely form the core secretome of rust pathogens. In contrast, 261 tribes were identified as lineage-specific, with 116 containing proteins specifically from *M. larici-populina* (a total of 544 secreted proteins) and 145 containing proteins only from *P. graminis* f. sp. *tritici* (a total of 431 secreted proteins).

Most tribes shared between species (∼80%, 138 tribes) could be annotated by similarity to known proteins, whereas lineage-specific tribes could rarely be annotated. Only 19.5% of lineage-specific tribes were annotated, with 15 *M. larici-populina*-specific and 36 *P. graminis* f. sp. *tritici*-specific tribes (**Figure 3B**). This is consistent with the distinction between a shared core secretome and lineage-specific protein innovations. The distribution of number of proteins in tribes was shifted towards higher number for core secretome tribes (**Figure 3C**). Approximately one half of the secretome of *M. larici-populina* and *P. graminis* f. sp. *tritici* constitute the putative core secreted protein set, grouped into shared annotated tribes. The remaining half contained tribes of non-annotated lineage-specific secreted proteins, which are likely to be enriched in effector candidates.

### The secretome of rust fungi is enriched in forty-seven PFAM domains

To document biological functions specifically enriched in the secretome of rust fungi, we mapped PFAM domains on the proteomes of the two species. We then applied the filtering module and tested for enrichment of each domain among secreted proteins versus non-secreted proteins. We identified 47 PFAM domains significantly enriched in the secretome of rust fungi, including 36 PFAM-A domains and 11 PFAM-B domains that lack annotation (**File S1**). The enriched PFAM-A domains were distributed among 45 protein tribes of rust fungi containing three or more members. Of these, 43 tribes (96%) contained proteins from both species (**File S1**). Tribe 234 was the only tribe specific to *M. larici-populina* and contained proteins with the PF01670 (xyloglucan-specific endo-beta-glucanase) PFAM domain. Tribe 422 containing the PF01161 (phosphatidylethanolamine binding protein) PFAM domain was specific to *P. graminis* f. sp. *tritici*. We hypothesize that proteins in tribes containing members from both species may perform core biological functions of secreted proteins and functions that may be unrelated to host specificity. In accordance, twenty-one PFAM domains (∼60% of the enriched PFAM-A) corresponded to typical secreted enzymes, such as proteases, plant cell wall degrading enzymes, phospholipases, and detoxification enzymes (**Table S1**).

Five domains (∼14% of the enriched PFAM-A) had previously been reported as involved in pathogenicity (**Table 1**). These include CFEM (PF05730, tribe 179), an eight-cysteine-containing domain unique to fungi [32], also identified in the *Melampsora* secretome [33]. The developmentally regulated MAPK interacting protein (DRMIP) domain (PF10342, tribe 132) found in fungal serine/threonine-rich membrane-anchored proteins homologous to HESP-379 from *M. lini* [34]. One member of the DRMIP family from the fungus *Lentinula edodes* that interacts with the kinase *LeMAPK* and is proposed to be involved in cell differentiation during the development of *L. edodes* [35]. The cysteine-rich secretory proteins, antigen 5, and pathogenesis-related 1 protein, CAP protein family (domain PF00188, tribe 83), are proposed to be calcium chelating serine proteases [36]. These proteins can function as proteases or protease inhibitors, ion channel regulators, tumour suppressors or pro-oncogenic proteins, and in cell-cell adhesion [37]. The thaumatin domain (PF00314, tribes 163 and 394) is found in pathogenesis-related (PR) proteins with antifungal activity. They are also involved in systematically acquired resistance and stress responses in plants, via unknown mechanisms [38]. In plant TLP-Ks, a thaumatin-like protein (TLP) domain is associated with a protein kinase domain and TLP-Ks were proposed to act as receptor-like kinases during defence responses [39], in particular against *M. larici-populina* [40]. Thaumatin-like secreted proteins of rust fungi may alter this plant-signalling pathway and have been reported in the *Melampsora* secretome [33]. The rare lipoprotein A domain (PF03330, tribe 52) adopts a double-psi beta-barrel fold, which is also found in the cerrato-palatin (CP) fungal elicitor protein [41]. CP induces a defence response in the plant and is therefore considered a pathogen-associated molecular pattern (PAMP) that has been proposed to be involved in polysaccharide recognition [41].

**Table 1:** Non-enzymatic PFAM domains significantly enriched in the secretome of rust fungi.

Pfam	Description	Enrich-ment^1^	p-value^2^	Total	Secreted^3^	Haustoria^4^	Tribes^5^
**Proteins with documented or suggested role in plant-pathogen interactions**							
**PF00188**	Cysteine-rich secretory protein family	**5.4**	1.94E-03	17	7	5	83 (x14)
**PF00314**	Thaumatin family	**6.6**	1.06E-02	10	5	3	163 (x5), 394 (x3)
**PF03330**	Rare lipoprotein A (RlpA)-like double-psi beta-barrel	**6.1**	4.81E-10	26	12	6	52 (x14)
**PF05730**	CFEM fungal specific cysteine rich domain	**10.1**	8.62E^−13^	17	13	16	160, 179 (x4)
**PF10342**	Developmentally Regulated MAPK Interacting Protein	**7.9**	2.26E-10	15	9	13	132 (x2)
**Atypical secretome functions**							
**PF01161**	Phosphatidylethanolamine-binding protein	**6.6**	1.04E^−07^	18	9	5	199 (x4), 248 (x4), 422 (x3)
**PF02221**	ML domain - MD-2-related lipid recognition domain	**11.0**	3.02E^−07^	6	5	0	363, 415 (x2)
**PF10281**	Putative stress-responsive nuclear envelope protein	**6.0**	4.43E^−02^	11	5	5	551 (x5)
**PF11327**	Protein of unknown function (DUF3129)	**9.2**	8.62E^−13^	23	16	4	57 (x12)

1 Enrichment: Number of PFAM hits in secretome over number of hits in non secreted proteins;

2 p-value for enrichment in secretome;

3 number of domains in secretome;

4 number of domains in haustorial proteins;

5 tribes containing at least two instances of the domain with number of instances in parenthesis.

Finally, some of the secretome-enriched domains we identified are atypical for secreted proteins (**Table 1**). The putative stress-responsive nuclear envelope protein domain (PF10281, tribe 551) is related to hydrophilins such as LEA1 that prevent aggregation of structurally compromised proteins [42]. The phosphatidylethanolamine-binding protein domain (PF01161, tribes 199, 248 and 422) is involved in lipid binding, serine protease inhibition and the regulation of several signalling pathways such as the MAP kinase pathway. Immunoglobulin-like domains such as the domain of unknown function DUF3129 (PF11327, tribe 57) could be involved in cell-cell recognition or cell-surface receptor signalling. Proteins containing the ML domain (PF02221, tribes 363 and 415) have been implicated in lipid recognition, particularly in the recognition of pathogen related products such as lipopolysaccharide (LPS) binding and signalling. LPS and glycoproteins have been detected in the neck region of haustoria [43]. These atypical PFAM domains enriched in secretome proteins might represent specific functions of the secreted proteins of rust fungi.

**Figure 4:**
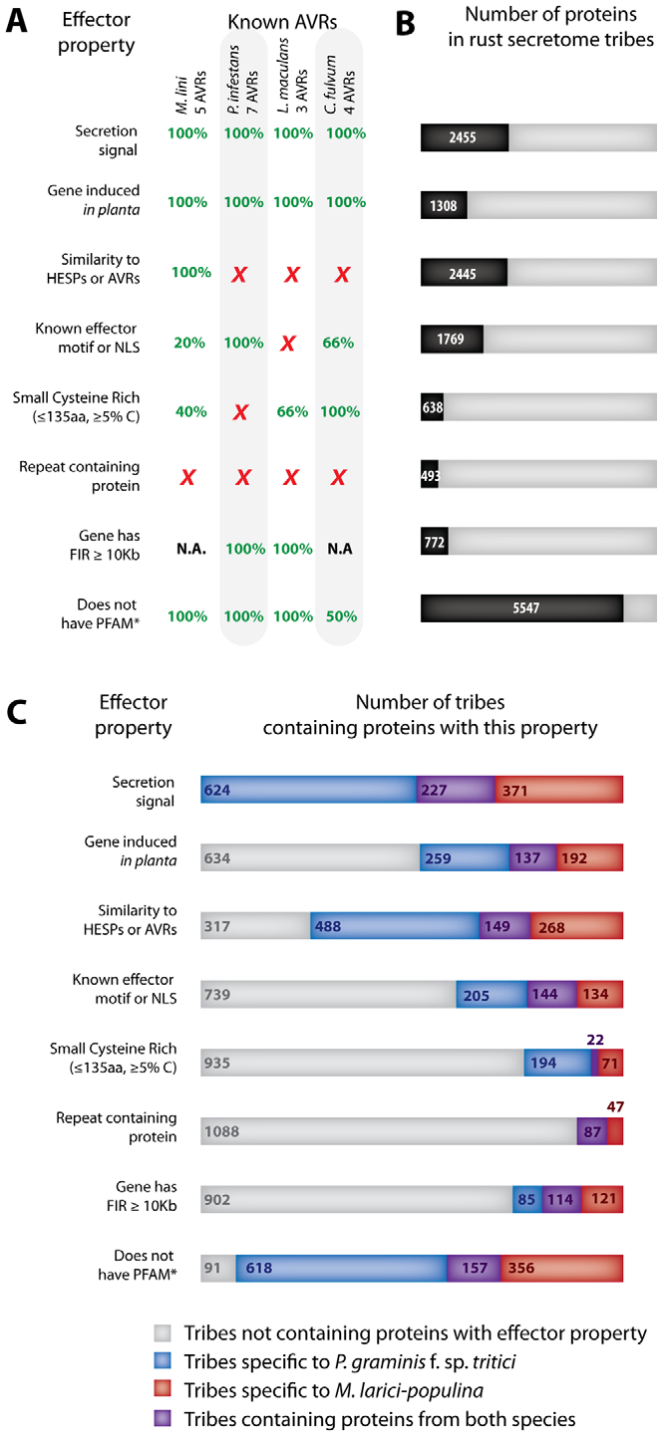
Distribution of effector features in the *M. larici-populina and P. graminis* f. sp. *tritici* secretome proteins and tribes. (A) Percentage of known avirulence effectors (AVRs) from *M. lini*, *P. infestans*, *L. maculans* and *C. fulvum* showing each effector property. A red cross indicates no match; N.A., not available. (B) Number of proteins grouped in secretome tribes showing each one of eight effector properties. Numbers on charts refers to total number of proteins. (C) The distribution of eight effector features in core and lineage-specific secretome tribes of rust fungi. *Five PFAM domains associated with pathogenicity and enriched in secretome tribes of rust fungi (Table 1) and PFAM-B domains were permitted.

In general, known effector proteins rarely contain PFAM domains [22], [44], [45]. We assessed nineteen AVR effectors for the presence of PFAM domains (**Figure 4A**). *C. fulvum* Avr4 had a significant hit to the Chitin Binding Module PFAM domain (PF03067) and Ecp6 to the LysM PFAM domain (PF01476), however these domains were not enriched in the secretome of rust fungi. The remaining validated AVR effectors considered in this study do not have significant similarities to known PFAM domains. Therefore, we considered that one effector property is the absence of a PFAM domain, with the exception of five domains that are associated with pathogenicity and enriched in the secretome of rust fungi (Table 1). Using this criterion, nearly 80% (5294) of the proteins analyzed did not harbour a PFAM domain (**Figure 4B**). A total of 1108 tribes contained at least one protein with no PFAM domain, of which 141 contained proteins from both rust fungal species (**Figure 4C**).

### *M. larici-populina* and *P. graminis* f. sp. *tritici in planta* induced genes

All known AVRs from *M. lini*, *L. maculans*, *C. fulvum*, and *P. infestans* are expressed *in planta* [22], [46]. We used published transcriptome data [27] to identify proteins that are encoded by genes induced at least 2-fold *in planta* when compared to resting urediniospores. We identified a total of 1308 proteins (19.6% of proteins analyzed), encoded by genes induced *in planta* at 96 hours post-inoculation (**Figure 4B**). These proteins were distributed among 847 tribes, of which 137 contained proteins from both species of rust fungi (**Figure 4C**).

### *M. larici-populina* and *P. graminis* f. sp. *tritici* proteins with similarity to haustorial ESTs

All five characterized *M. lini* AVRs were originally identified from cDNA sequences prepared from purified haustoria (**Figure 4A**). We therefore searched for proteins of the secretome of rust fungi showing similarity to available rust fungi haustorial ESTs from *P. triticina*, *P. striiformis* f. sp. *tritici* [47], *M. larici-populina* [33], and *M. lini* [34]. We identified 2445 proteins with similarity to haustoria expressed secreted proteins (HESPs) or fungal AVRs (**Figure 4B**). These proteins were distributed across 905 tribes, of which 149 contained proteins from both species of rust fungi (**Figure 4C**). Notably, some putative haustorial proteins showed similarity to the known flax rust pathogen AVR proteins AvrL567, AvrP123, AvrM and AvrP4 (**File S2**).

### The secretome of rust fungi contains proteins with motifs common to filamentous plant pathogen effectors

We used nuclear localization signals (NLS) and effector signature motifs [48], [49], [50], [51], such as the Y/F/WxC motif found in *M. lini* AvrL567 and *C. fulvum* Avr2 and Avr4, as a criterion for mining effectors from the secretome of *M. larici-populina* and *P. graminis* f. sp. *tritici*. We identified 1769 proteins with either a reported effector motif or an NLS (**Figure 4B**). The most abundant motif was Y/F/WxC [30] that was identified in 999 secreted proteins distributed across 340 tribes. In total, proteins with effector motifs or NLS were distributed across 483 tribes, of which 144 contained proteins from both species of rust fungi (**Figure 4C**).

### Some genes of the secretome of rust fungi show unusually long intergenic distances

To identify candidate effectors based on the length of their flanking intergenic regions (FIRs) we calculated 5′ and 3′ FIRs for every gene in the *M. larici-populina* and *P. graminis* f. sp. *tritici* genomes. We sorted genes into two-dimensional data bins for each genome, as described earlier [46] (**Figure S1A**). This representation showed an overall expansion of *M. larici-populina* intergenic regions compared to *P. graminis* f. sp. *tritici*, with a significant proportion of genes having FIRs significantly longer than 10 Kb. However, the patterns produced by this method did not reveal groups of genes with dramatically extended FIRs compared to median values, in contrast to *L. maculans* [21] or *P. infestans* [46] where effector genes often have long FIRs.

To determine whether secretome genes showed a distinctive pattern in the distribution of the length of their FIRs we analyzed the enrichment of secretome genes along the bins, as a ratio of the frequency in a bin compared to the frequency in the whole genome (**Figure S1B**). We found that globally, genes with FIRs less than ∼800 bp tended to be depleted for secretome genes whereas genes with at least one FIR longer than ∼10 Kb were enriched (**Figure S1B**), particularly in the *M. larici-populina* genome. Therefore, we considered having a FIR longer than 10 Kb as a criterion for selecting candidate effector genes. Tribes containing a high proportion of genes with FIR>10 Kb tended to be small, non-annotated and contained a high proportion of genes encoding secreted proteins (**Figure S1C**), which was expected for candidate effector tribes. We identified 772 secretome genes with FIR>10 Kb (**Figure 4B**), distributed across 320 tribes (**Figure 4B**). One hundred and fourteen of these tribes contained proteins from both species rust of rust fungi. In the absence of genome sequences for the corresponding species, information on FIRs for *M. lini* and *C. fulvum* AVRs could not be calculated. However, all *P. infestans* and *L. maculans* AVR genes have at least one intergenic region longer than 10 Kb (**Figure 4A**). In contrast to what has been reported for *P. infestans*, *B. graminis* and *L. maculans*, long intergenic distances do not seem to be a common feature of effector genes of rust fungi. Nevertheless, some genes of the secretome of rust fungi showed unusually long intergenic distances and may, therefore, correspond to a specific subset of fast-evolving effector candidates of rust fungi.

### Families of small secreted cysteine-rich proteins in the secretome of rust fungi

Filamentous plant pathogen effectors are often small cysteine-rich (SCR) proteins [22]. Based on the published examples in **Table S2**, known SCR effectors are typically less than 150 amino acids long and have a cysteine content higher than 3%. To identify candidate SCR effector tribes in the secretome of *M. larici-populina* and *P. graminis* f. sp. *tritici* we calculated the cysteine content and length of each protein. Tribes containing at least one secreted protein shorter than 150 amino acids and with a cysteine content higher than 3% were considered as SCR tribes. We found a total of 638 SCRs proteins in the secretome of *M. larici-populina* and *P. graminis* f. sp. *tritici* (**Figure 4B**), which contributed to 287 tribes (**Figure 4C**). Remarkably, only 22 tribes containing SCRs included proteins from both species of rust fungi (**Figure 4C**), suggesting a high divergence of cysteine patterns in these proteins. The presence of SCR tribes in these secretome is consistent with the results from *de novo* motif searches that identified conserved cysteine motifs shared across several tribes (**Figure 2**).

### The secretome of *M. larici-populina* but not *P. graminis* f. sp. *tritici* includes repeat-containing proteins (RCPs)

To identify tribes containing RCPs in the secretome of *M. larici-populina* and *P. graminis* f. sp. *tritici* we used the T-REKS algorithm [52] to systematically search for tandem repeats in proteins from secretome tribes. We found a total of 493 RCPs (**Figure 4B**), which contributed to 134 tribes (**Figure 4C**). Surprisingly, all RCPs belonged to the *M. larici-populina* proteome. Although some *M. larci-populina* RCPs were grouped in tribes with proteins from both species, the *P. graminis* proteins in these tribes were not classified as RCPs. An *M. larici-populina* gene encoding a pentatricopeptide repeat-containing protein was identified previously as specifically induced in the sporogenous area of an infected poplar leaf [53], supporting the importance of RCPs during the infection process.

### Ranking candidate effectors by hierarchical clustering of tribes

**Figure 5:**
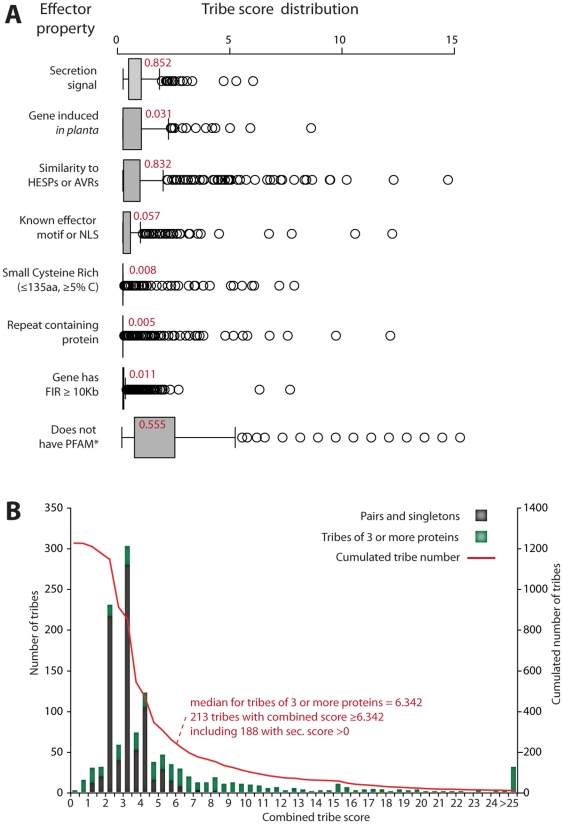
Distribution of scores for effector candidates in secretome tribes from *M. larici-populina* and *P. graminis* f. sp. *tritici*. (A) The distribution of scores (equivalent to -log of e-value) for individual effector properties among tribes given as a boxplot (same conventions as in Figures 2 and 3). The median value is indicated in red. (B) Distribution of combined scores (sum of scores for individual effector properties) among the 1222 tribes analyzed. A combined score threshold ≥6.342 (median value for tribes of 3 or members) and secretion signal (sec.) score >0 was used to select 188 tribes for hierarchical clustering. *Five PFAM domains associated with pathogenicity and enriched in secretome tribes of rust fungi (Table 1) and PFAM-B domains were permitted.

**Figure 6:**
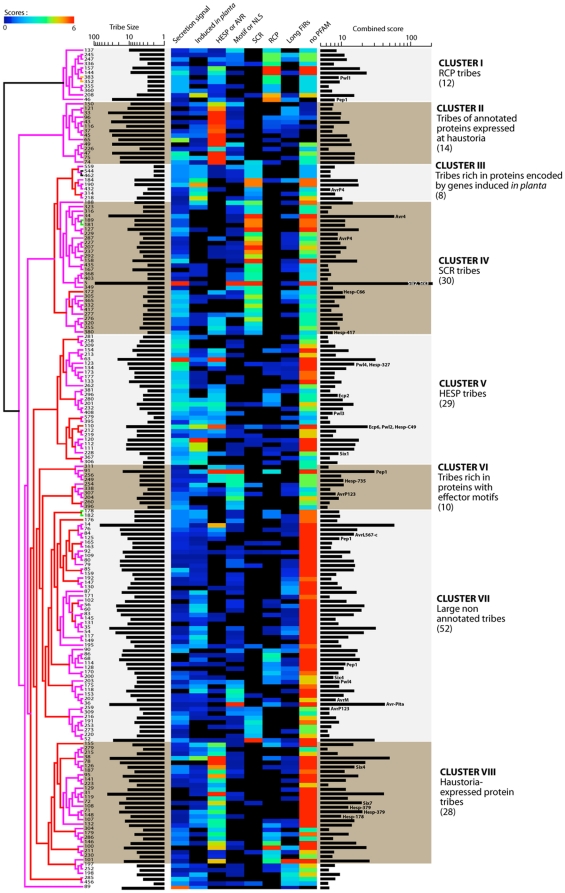
Hierarchical clustering of the secretome reveals clusters of secreted protein families as high priority effector candidates. A complete hierarchical cluster tree of the 188 secretome tribes with combined score ≥6.342 and secretion signal score >0. The tribe identifiers are indicated at the tip the branches of the boostrap support tree. For each tribe, the number of proteins is indicated on the left of the clustering image and the combined score on the right. When proteins in a tribe show similarity (10e^−5^ BlastP e-value threshold) to fungal AVRs and *M. lini* HESPs, these are indicated along the score bars. We distinguished eight clusters. The number of tribes in a cluster is indicated in parenthesis. AVR, avirulence protein; HESP, haustoria expressed secreted protein; FIR, flanking intergenic region; NLS, nuclear localization signal; RCP, repeat containing protein; SCR, small cysteine rich protein.

We noted a variable and complex distribution of the effector properties we analyzed across proteins and tribes. A given effector property may match only a subset of proteins within a tribe, while multiple effector properties may match a single protein in a tribe. Therefore, tribe content in relation to matching effector properties can be considered as a set of quantitative data amenable to classification by methods such as hierarchical clustering, which in biology is typically used for classification of gene expression data. To reduce bias due to the variable size of tribes, we associated an e-value to each effector property for every tribe examined. The e-value corresponds to the likelihood of obtaining at least the same number of proteins with the given property by chance (see methods). These e-values were log-converted into a score (**Figure 5A**), and a combined score was calculated as the sum of scores associated to each effector property. The median combined score for tribes of three or more proteins was 6.342. A total of 213 tribes out of 1222 tribes examined had a combined score ≥6.342 (**Figure 5B**). Tribes that scored below this cut-off were omitted from further analysis, as they were frequently smaller tribes (pairs and singletons) more prone to harbouring effector properties by chance. In addition, considering that being secreted is a property that should be found in all effectors, we excluded 25 tribes (remaining was 188 tribes) with a secretion signal score of 0 out of the 213 that passed the combined score threshold. Next, we used hierarchical clustering to classify the 188 secretome tribes that passed the score threshold, based on the score associated to each of the eight effector properties (**File S2**). A hierarchical tree was built for the tribe variable, whereas the order of effector properties was not optimized by the algorithm but set manually by priority for enrichment in (i) presence of a secretion signal, (ii) *in planta* induced genes, (iii) similarity to haustorial proteins, (iv) presence of an effector motif or a NLS, (v) SCR proteins, (vi) RCPs, (vii) long FIR genes, (viii) proteins without PFAM domains (with the exception of the five PFAM domains associated with pathogenicity and PFAM-B domains that do not have functional annotations). The optimized hierarchical tree of tribes obtained after 1000 bootstrap rounds produced eight delimited clusters of tribes (**Figure 6** **, File S3**).

The hierarchical clustering approach allowed us to classify and sort a complex data set. Ranking tribes based on the prevalence of effector features highlighted tribes in clusters I, III, IV and V as the most likely to contain effectors because they fulfil most of the high value criteria. Tribes in cluster II contained mostly annotated proteins expressed in haustoria and likely contain proteins involved in core haustoria biological processes. Generally, tribes in clusters VI, VII and VIII contain a low number of secreted proteins that grouped with a large proportion of non-secreted proteins and are less likely to be *bona fide* families of secreted proteins.

The 12 tribes in cluster I contain a high number of RCPs. Tribe 383 contains proteins with similarity to *M. oryzae* Pwl1 and tribe 46 contains proteins with similarity to Pep1, a secreted effector from *Ustilago maydis* [54]. Tribes 144 and 208 had the highest combined score in this cluster.

Cluster III contains 8 tribes rich in *in planta* induced genes. Most of these tribes also obtained good scores for the presence of a secretion signal. Tribe 432 contains homologs of *M. lini* AvrP4. Tribes 184 and 190 obtained the highest combined score in this cluster.

The 30 tribes in cluster IV contain a high percentage of secreted proteins that consist mostly of SCR proteins. Compared to proteins in Cluster V, they have a lower incidence of similarity to haustorial proteins, indicating that they may not be secreted from haustoria. Of the SCR-containing tribes, tribe 34 is similar to *C. fulvum* chitin-binding Avr4 effector, tribe 287 is similar to AvrP4 from *M. lini*, tribes 372 and 380 show similarity to uncharacterized *M. lini* HESPs, and tribe 5 corresponds to the largest SCR tribe from *M. larici-populina* reported in [27]. Tribe 5 and 34 obtained the highest combined score in this cluster.

Cluster V consists of 29 tribes that contain a high proportion of predicted secreted proteins and proteins with similarity to haustorial proteins. They corresponded to the HESP tribes of the *M. larici-populina* and *P. graminis* f. sp. *tritici* proteomes. Tribe 110 in cluster II contains proteins similar to Pwl2, an Avr effector from *M. oryzae* [15] and *C. fulvum* Ecp6 LysM domain virulence effector. Tribes 123 and 408 contain proteins similar to *M. oryzae* Pwl4 and Pwl3 respectively. Tribe 228 contains with similarity to *C. fulvum* Six1 (Avr3) and tribe 381 proteins similar to *C. fulvum* Ecp2. Some of these tribes also contain a large number of *in planta* induced genes suggesting they are good effector candidates. Tribes 63 and 110 had the highest combined score in this cluster.

Clusters II, VI, VII and VIII have a lower probability of including effector candidates. In addition to the 14 tribes in cluster II, likely involved in core haustoria biological processes, three clusters (VI, VII and VIII) contain tribes with generally low scores for the most important effector properties, in particular a low content in proteins predicted to be secreted. The 28 tribes in cluster VIII have high scores for proteins with similarity to haustorial proteins, and the absence of annotated proteins, but low scores for the number of proteins predicted to be secreted. Similarly, the 52 tribes in cluster VII have low scores for their content in secreted proteins, in proteins encoded by *in planta* induced genes and in proteins with similarity to haustorial proteins. Although they had high score for the presence of effector motifs or NLS, the 10 tribes of cluster VI have low scores for their content in proteins predicted to be secreted, encoded by *in planta* induced genes, and with few exceptions for proteins with similarity to haustorial proteins. For these reasons, we decided to rank clusters VI, VII and VIII with a lower priority. Nevertheless, five tribes in cluster VIII (tribes 78, 108, 71, 148 and 107), eight tribes in cluster VII (tribes 84, 125, 114, 200, 203, 202, 36 and 259) and three tribes in cluster VI (tribes 91, 249, and 307) contained proteins with similarity to fungal effectors or reported *M. lini* HESPs, and might therefore constitute interesting effector candidates. The unexpected clustering of these tribes might result from inaccurate gene models, preventing accurate signal peptide prediction, unspecific aggregation in a tribe, or spurious similarity to effectors.

### A selection of remarkable candidate effector tribes of rust fungi

To identify the tribes with the highest likelihood of containing effector proteins, we selected the two tribes with the highest combined score from the four most promising clusters identified above (Clusters I, III, IV and V) (**Table 2**). We propose these eight tribes as including high-priority candidate effectors and we examined them in more detail.

**Table 2:** A selection of 8 tribes of candidate effectors of rust fungi.

HCL cluster	Tribe n°	Comb. Score^1^	Size^2^	Secreted^3^	Haus-toria^4^	Ind. in planta^5^	Species distribution	Annotation	Similarity	Comments
**I**	**144**	23.4	9	8(2.7)	8(2.7)	1(0.1)	*M. larici* only	9 Gly-rich RCPs (10.3), 2 long FIRs (0.5)	Uncharacterized *P. triticina* HESPs	
**I**	**208**	15.5	6	3(0.4)	6(4.4)	3(0.4)	*M. larici* only	5 Gly-rich RCPs (4.9)	Uncharacterized *P. triticina* HESPs	
**III**	**190**	17.9	7	6(1.9)	1(0)	7(5.1)	*P. graminis* only	6 SCRs (5.3)	NA	. Genes clustered in *P. graminis* genome
**III**	**184**	17.4	7	5(1.2)	0(0)	6(3.5)	*M. larici* only	6 SCRs (5.3), 3 with long FIRs (1.4)	NA	
**IV**	**5**	210.3	92	62(8.7)	7(0)	13(0)	*M. larici* only	90 classified as SCRs (90.8), 12 with long FIRs (0.4)	*F. oxysporum* Six2, Six3, *M. oryzae* Avr-Pita	Described in Duplessis *et al*. 2011. 2 genes in Top100 induced in germinating urediniospores^a^
**IV**	**34**	58.8	38	20(1.4)	4(0)	3(0)	*M. larici* only	32 SCRs (26.7), 7 with long FIRs (0.8)	*C. fulvum* Avr4	
**V**	**63**	31.3	21	19(6.3)	20(7.6)	7(1.0)	*P. graminis* only	NA	NA	. 4 genes in Top100 induced in infected wheat^b^; 1 gene in Top100 induced in germinating urediniospores^c^
**V**	**110**	24.2	12	10(2.9)	12(5.6)	9(4.3)	6 *M. larici*6 *P. graminis*	3 SCRs (1.0), 3 genes with long FIRs (0.8), 1 RCP (0.2)	*M. lini* HESP-C49, *C. fulvum* Ecp6, *M. oryzae* Pwl2	. 2 *M. larici* genes in Top100 induced in infected poplar leaves^d^; 3 *P. graminis* genes in Top100 induced in infected wheat^b^

1 Combined score;

2 Number of proteins in tribe;

3 number of secreted proteins in tribe;

4 number of haustorial proteins in tribe;

5 number of proteins encoded by *in planta* induced genes. Values in parenthesis show scores obtained for each property. FIR, Flanking Intergenic Regions; NA; Not Applicable; RCP, Repeat-Containing Protein; SCR, Small Cysteine Rich protein. Tables from Duplessis *et al.* 2011 cited for gene expression:

a Table S11;

b Table S14;

c Table S15;

d Table S10.

Tribe 144 had the highest combined score (23.4) in cluster I. It contains 9 RCPs with Glycine-rich repeats, 8 proteins predicted to be secreted and 8 with similarity to haustorial proteins. Tribe 208 obtained the second highest combined score from cluster I (15.5), because it contains 6 proteins with similarity to haustorial proteins and 5 Glycine-rich RCPs. It only contains 3 secreted proteins, and 3 encoded by *in planta* induced genes, suggesting that few copies of genes from this family may be effectors. These two RCP tribes are specific to *M. larici-populina*.

Tribe 190 had the highest score in cluster III (17.9). It contains seven *P. graminis* f. sp. *tritici* proteins, all predicted to be secreted and induced *in planta*. Six of these have features of SCR proteins and genes encoding tribe 190 proteins are clustered within the *P. graminis* f. sp. *tritici* genome. Tribe 184 had the second highest score in cluster III (17.4), it contains seven *M. larici-populina* proteins, five of which were predicted to be secreted, six induced *in planta*, and six with SCR features.

The highest scoring tribe in cluster IV was tribe 5 (210.3), a large tribe (92 proteins) of *M. larici-populina*-specific SCR proteins. Among those, 67 were predicted to be secreted, 13 induced *in planta* at least 2-fold and 7 are similar to haustorial proteins. Tribe 5 proteins are also similar to *F. oxysporum* Six2 and Six3 cysteine-rich effectors. This tribe corresponds to the small secreted protein family described previously in Duplessis *et al.* [27]. Tribe 34 had the second highest score (58.8) in cluster IV. It contains 38 *M. larici-populina* proteins, 20 of which were predicted to be secreted and 32 have SCR features. Four proteins from tribe 34 are similar to *C. fulvum* Avr4 cysteine-rich effector.

Tribe 63 has the highest combined score in Cluster V (31.3). This tribe was specific to *P. graminis* f. sp. *tritici* and contains 21 proteins of which 19 have signal peptides and 20 have high similarity to uncharacterized HESPs. Seven members of this tribe were upregulated higher than 2-fold during infection suggesting that they may play a role at the plant-pathogen interface, potentially as effectors. Tribe 110 had the second highest combined score (24.2) in cluster II. Out of 12 proteins in this tribe, 10 have a secretion signal, 9 were induced *in planta*, and all have homology to haustorial proteins. In particular, homologs of *M. lini* HESP-C49, *C. fulvum* Ecp6 and *M. oryzae* Pwl2 were found in this tribe. Whereas most top-scoring tribes highlighted here are lineage-specific, tribe 110 contains 6 proteins from *M. larici-populina* and 6 from *P. graminis* f. sp. *tritici*.

### Conclusions

In this study, we report a bioinformatic pipeline aimed specifically at finding effector genes from two agriculturally important rust fungi whose genome sequences have recently been released. Our pipeline revealed a list of candidate effector genes that constitute a valuable resource for accelerating the discovery and deployment of genetic resistance to rust fungi. To date, only a few attempts have been made to comprehensively characterize the secretome of rust fungi. Joly *et al.* [33] analyzed the secretome of four *Melampsora* species and identified thirteen protein families showing evidence of positive selection [33]. The Joly *et al.* [33] study provides an additional selection criterion for effector mining that complements our analysis. More recently, Duplessis *et al.* [27] probed the genome sequence of *M. larici-populina* and *P. graminis* f. sp. *tritici* for small secreted proteins [27]. Unlike the analyses by Duplessis *et al* [27], we processed secreted proteins of all sizes and based our protein clustering on the combined secretome of both species. We also omitted signal peptide sequences from our clustering analysis to classify the secretome based on the functional domains of the candidate effectors. We included non-secreted proteins in the Markov clustering approach in order to detect false positives and to use tribe enrichment in secreted proteins as a ranking criterion. Finally, we developed an original hierarchical clustering method to integrate multiple effector mining criteria to classify and rank tribes based on their likelihood of being genuine effectors. Hierarchical clustering of effector features resulted in a priority list that should prove valuable for follow-up wet lab experiments. These could include functional expression of the candidate genes in resistant plant genotypes to identify effectors with avirulence activity.

## Methods

### Secretome prediction and annotation

Predicted proteomes of *M. larici-populina* and *P. graminis* f. sp. *tritici* were obtained from [55] and [56] respectively. The secretomes were defined using PexFinder [57]. Transmembrane domain containing proteins and proteins with mitochondrial signal peptides were removed using TMHMM and TargetP, respectively. Automated BlastP-based annotation was performed on proteins included in the secretome tribes of rust fungi using Blast2GO [58] with default parameters. In addition, a database including *P. striiformis* f. sp. *tritici* haustoria ESTs [59], *P. triticina* haustoria ESTs [47], *M. lini* HESPs [34], fungal AVRs [22], and *M. larici-populina* haustoria ESTs [33] was constructed. A BlastP analysis of proteins included in the secretome of rust fungi was conducted using this haustorial EST database, with an e-value cutoff of 10^−5^. We searched each protein for the effector motifs [L/I]xAR [60], [61], [R/K]CxxCx12H [60], RxLR [9], [Y/F/W]xC [30], YxSL[R/K] [62] and G[I/F/Y][A/L/S/T]R [34] between amino acids 10 to 110 using Perl scripts. Nuclear localisation signals were predicted with PredictNLS [63]. Protein internal repeats were predicted using T-Reks [52]. Disulfide bridges were predicted using Disulfind [64].

### Markov clustering

Signal peptide regions were removed from all secreted proteins and then the truncated proteins used in a similarity search against a combined database of the two proteomes of rust fungi (e-value 10^−5^). Proteins were clustered using TribeMCL [65] following methods described in [46].

### PFAM enrichment analysis

PFAM domains were mapped on proteins of the secretome tribes using the PFAM batch search server [66]. Domain hits with e-values higher than 10^−5^ were ignored. We considered domains as enriched when their frequency (number of domains per protein) in the secreted proteins was higher than their frequency among non-secreted proteins; domains were considered as depleted otherwise. Statistical significance of the enrichment ratios was assessed using a Chi-square test with Bonferroni correction. Enrichment and depletion were considered significant when p-value<0.01.

### *De novo* motif searches

*De novo* protein motif search was performed on proteins in secretome tribes using MEME [28]. The program was set to report the 25 most robust motifs of 4 to 10 amino-acids, occurring zero or once per sequence, among the proteins belonging to tribes of three or more members. These motifs were classified based on the dispersion of their position along the protein sequence, and the number of tribes in which they were found. Motifs showing reduced dispersion (interquartile range for motif position <10 amino acids) and found in at least two proteins in each of at least 3 different tribes were considered as conserved and reported.

### Scoring and tribes ranking

An e-value was associated to the tribes for each of the eight effector properties analyzed. This e-value was calculated based on the probability of randomly grouping the same number of proteins with a given effector property as a tribe, from the complete set of analyzed proteins. This probability was calculated as follows. Let k be the number of proteins matching a given effector property in a tribe; n be the size of that tribe; K be the total number of proteins matching a given effector property among grouped proteins and N be the total number of proteins grouped in a tribe. The number of tribes of size n with k proteins matching an effector property is the number of k-combinations from a set of n elements, given by the formula:
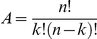
equivalent to 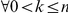


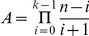
and A = 1 for k = 0. The probability of having exactly k proteins matching a given effector property in a tribe of n proteins is BxC, where B is the probability of having k proteins matching a given effector property and C is the probability of having n−k proteins not matching that property.

So 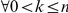


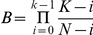
And B = 1 for k = 0

And 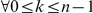


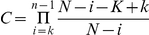
Therefore the likelihood of having a tribe of n proteins with k proteins matching a given effector property is P(k) = AxBxC. We defined the e-value associated with a given effector property and a given tribe as
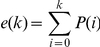
the corresponding score as −log_10_(e(k)), and the combined score as the sum of scores obtained for each effector property by a given tribe. These scores were calculated using homemade scripts in R.

### Hierarchical clustering

The hierarchical clustering analysis was conducted using MEV4 [67]. The 177 secretome tribes with score ≥6 were considered as ‘genes’, each of the eight effector properties (being secreted, induced *in planta*, having similarity to haustorial proteins, SCR properties, RCP properties and no PFAM annotation) were considered as ‘samples’. The score associated to each property for proteins in tribes were considered as ‘intensity’ values. The tribe hierarchical tree was optimized using 1000 bootstrap runs with Pearson correlation coefficient as distance value, and average linkage between groups. The priority of effector properties used for clustering was set manually and ranked as described in the text.

## Supporting Information

Figure S1 **Analysis of genome architecture used to define the threshold for long FIR genes.** (A) Distribution of *P. graminis f. sp. tritici* and *M. larici-populina* genes according to the length of their FIRs. Genes were sorted into two-dimensional data bins for each genome and number of genes is shown by a colour code. Crosses indicate median value for the two genomes combined; dotted circles are given as a reference to compare the two genomes; arrows point toward areas of the graph illustrating an overall expansion of *M. larici-populina* intergenic regions compared to *P. graminis* f. sp. *tritici*. (B) Same diagrams as in A showing the ratio of the frequency of secretome genes in a bin compared to frequency in the whole genome. Genes with FIRs less than ∼800 bp tend to be depleted in secretome genes (green dotted line) whereas genes with at least one FIR longer than ∼10 Kb tended to be enriched in secretome genes (purple dotted line). (C) Distribution of secretome tribes according to their content in secreted proteins (Y-axis) and in proteins encoded by genes with at least one FIR longer than 10 Kb (X-axis). Size of bubbles corresponds to size of the tribes.
(PDF)

Table S1 **Typical secreted enzyme PFAM domains enriched in the secretome of rust fungi.** Table providing enrichment fold, p-value of a chi-squared test for enrichment in secretome, number of total and secreted proteins and list of tribes containing the PFAM domains.
(DOC)

Table S2 **List of known small-secreted cysteine rich proteins (SCRs) used to define SCR features, with associated references.** Microsoft Excel worksheet.
(XLS)

File S1 **Complete list of PFAM domains and Gene Ontology terms detected in the proteomes of rust fungi and secretome enrichment analysis.** Microsoft Excel Workbook containing thirteen worksheets with the complete list of PFAM domain and Gene Ontology terms found in the secretome of rust fungi, enrichment values, P-value of chi-square test for enrichment with Bonferroni correction, and list of tribes containing the domains/ontologies. “Combined” secretome refers to analyses performed on the proteomes of *M. larici-populina* and *P. graminis* f. sp. *tritici* merged together. Analyses performed on separate proteomes from the two species are also provided.
(XLS)

File S2 **Complete list of tribes with full annotation data and features matching.** Microsoft Excel workbook containing (i) the list of proteins included in the tribe analysis with full annotation including effector properties, and (ii) the list of tribes with the number of proteins matching effector properties they contain.
(XLS)

File S3 **Details of the complete hierarchical cluster tree presented in** **Figure 4** **.**
(TXT)
