# Supplementary figures and images for: Using Hierarchical Clustering of Secreted Protein Families to Classify and Rank Candidate Effectors of Rust Fungi
Source: PLoS One. 2012 Jan 6;7(1):e29847. doi: 10.1371/journal.pone.0029847 (PMC3253089; doi:10.1371/journal.pone.0029847)

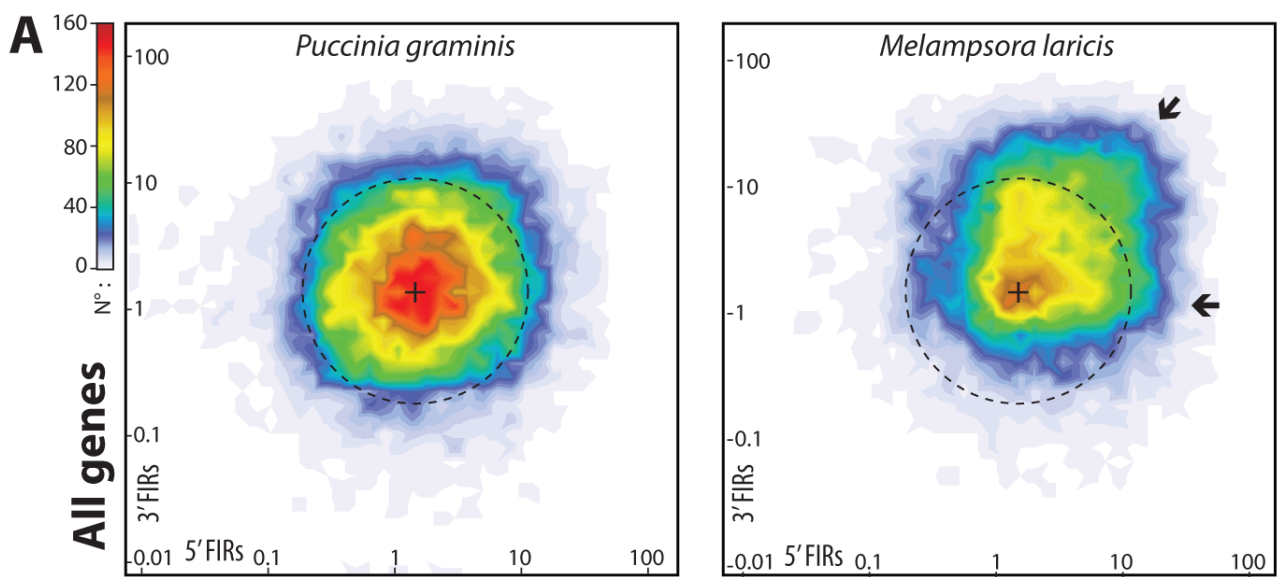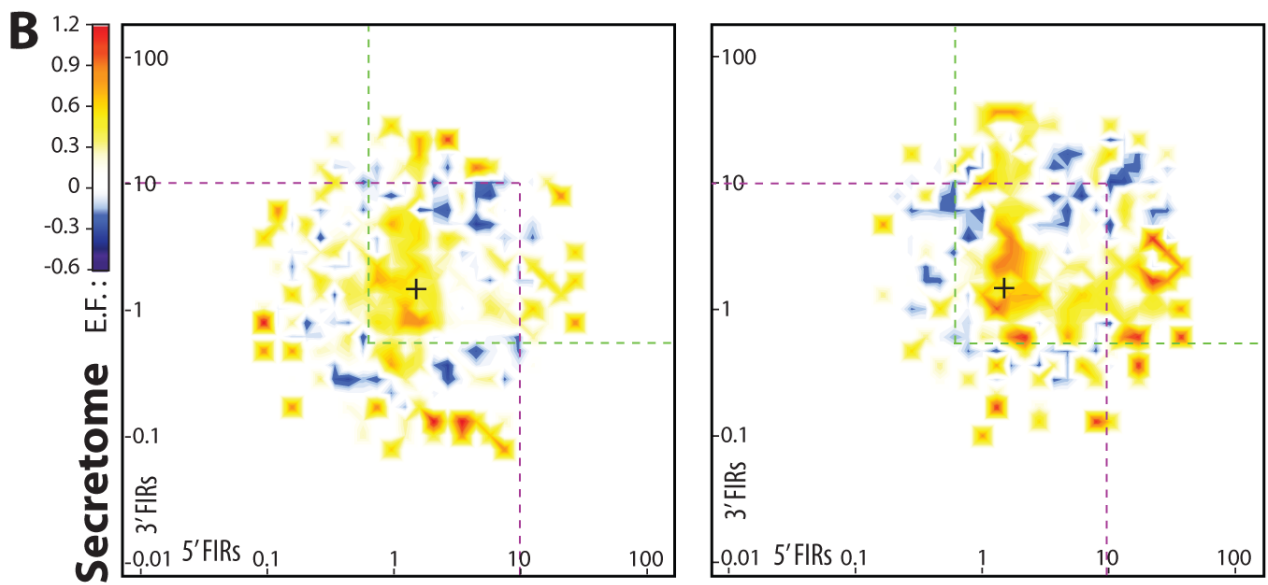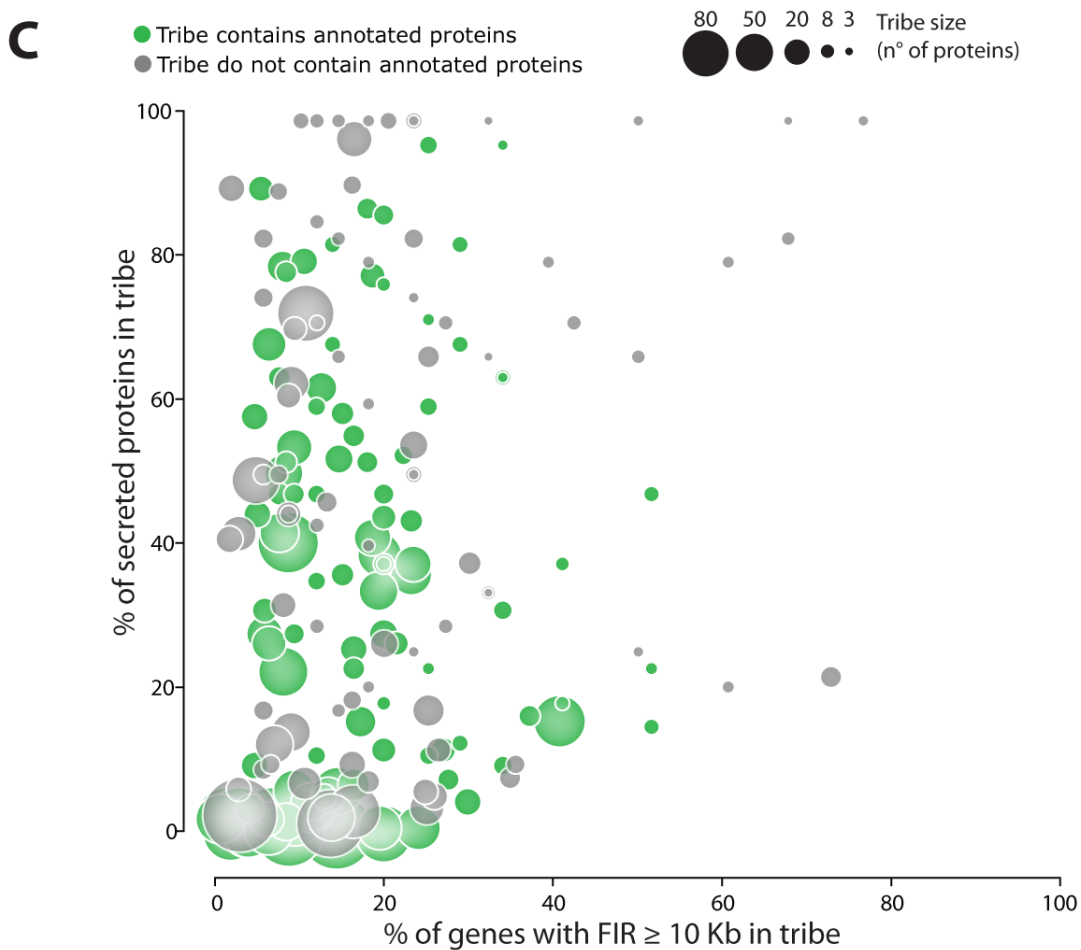

Supplement: Figure S1 — Analysis of genome architecture used to define the threshold for long FIR genes. (A) Distribution of P. graminis f. sp. tritici and M. larici-populina genes according to the length of their FIRs. Genes were sorted into two-dimensional data bins for each genome and number of genes is shown by a colour code. Crosses indicate median value for the two genomes combined; dotted circles are given as a reference to compare the two genomes; arrows point toward areas of the graph illustrating an overall expansion of M. larici-populina intergenic regions compared to P. graminis f. sp. tritici. (B) Same diagrams as in A showing the ratio of the frequency of secretome genes in a bin compared to frequency in the whole genome. Genes with FIRs less than ∼800 bp tend to be depleted in secretome genes (green dotted line) whereas genes with at least one FIR longer than ∼10 Kb tended to be enriched in secretome genes (purple dotted line). (C) Distribution of secretome tribes according to their content in secreted proteins (Y-axis) and in proteins encoded by genes with at least one FIR longer than 10 Kb (X-axis). Size of bubbles corresponds to size of the tribes. (PDF) [file pone.0029847.s001.pdf]
