# Supplementary material for: Using Hierarchical Clustering of Secreted Protein Families to Classify and Rank Candidate Effectors of Rust Fungi
Source: PLoS One. 2012 Jan 6;7(1):e29847. doi: 10.1371/journal.pone.0029847 (PMC3253089; doi:10.1371/journal.pone.0029847)
Supplement: Table S1 — Typical secreted enzyme PFAM domains enriched in the secretome of rust fungi. Table providing enrichment fold, p-value of a chi-squared test for enrichment in secretome, number of total and secreted proteins and list of tribes containing the PFAM domains. (DOC) [file pone.0029847.s002.doc]

**Additional File 2. Typical secreted enzyme PFAM domains significantly enriched in rust fungi secretomes.**

| Pfam | Description | **Enrich-ment ^1^** | p-val.^2^ | Total | Secreted ^3^ | Haustoria ^4^ | Tribes ^5^ |
| --- | --- | --- | --- | --- | --- | --- | --- |
| **PF00026** | Eukaryotic aspartyl protease | **4.4** | 7.83E-07 | 42 | 14 | 33 | 43 (x28), 156 (x2) |
| **PF00080** | Copper/zinc superoxide dismutase (SODC) | **7.9** | 8.62E-13 | 25 | 15 | 19 | 65 (x15) |
| **PF00082** | Subtilase family | **5.9** | 2.00E-09 | 27 | 12 | 2 | 88 (x10), 193 (x3), 238 (x3) |
| **PF00135** | Carboxylesterase | **5.3** | 1.18E-09 | 35 | 14 | 10 | 39 (x21) |
| **PF00150** | Cellulase (glycosyl hydrolase family 5) | **4.4** | 5.25E-04 | 30 | 10 | 1 | 73 (x8), 105 (x11) |
| **PF00703** | Glycosyl hydrolase family 2 | **8.2** | 2.09E-04 | 8 | 5 | 0 | 124 (x5) |
| **PF00704** | Glycosyl hydrolases family 18 | **6.0** | 8.62E-13 | 35 | 16 | 31 | 45 (x26) |
| **PF00722** | Glycosyl hydrolases family 16 | **6.6** | 1.06E-02 | 10 | 5 | 10 | 136 (x6) |
| **PF00840** | Glycosyl hydrolase family 7 | **12.5** | 8.62E-13 | 18 | 17 | 1 | 89 (x11) |
| **PF01095** | Pectinesterase | **8.1** | 3.69E-09 | 13 | 8 | 0 | 115 (x10) |
| **PF01522** | Polysaccharide deacetylase | **5.7** | 7.04E-12 | 35 | 15 | 27 | 37 (x27) |
| **PF01565** | FAD binding domain | **5.6** | 1.17E-07 | 26 | 11 | 2 | 64 (x9) |
| **PF01670** | Glycosyl hydrolase family 12 | **7.4** | 2.26E-09 | 16 | 9 | 2 | 24 (x5) |
| **PF01735** | Lysophospholipase catalytic domain | **4.9** | 1.26E-12 | 51 | 19 | 40 | 33 (x44) |
| **PF02065** | Melibiase (Alpha-galactosidase) | **7.3** | 1.85E-03 | 9 | 5 | 4 | 147 (x4), 186 (x3) |
| **PF02837** | Glycosyl hydrolases family 2, sugar binding domain | **7.2** | 1.00E-04 | 11 | 6 | 0 | 124 (x5), 206 (x3) |
| **PF03663** | Glycosyl hydrolase family 76 | **8.4** | 5.88E-08 | 11 | 7 | 0 | 138 (x10) |
| **PF07732** | Multicopper oxidase | **3.9** | 9.90E-02 | 27 | 8 | 16 | 24 (x19) |
| **PF08031** | Berberine and berberine like | **8.8** | 8.62E-13 | 15 | 10 | 1 | 64 (x6) |
| **PF10282** | 3-carboxy-cis,cis-muconate lactonizing enzyme | **6.6** | 5.87E-04 | 12 | 6 | 3 | 122 (x8) |
| **PF12222** | Peptide N-acetyl-beta-D-glucosaminyl asparaginase amidase A | **8.2** | 2.09E-04 | 8 | 5 | 0 | 162 (x7) |

^1^ Enrichment: Number of PFAM hits in secretome over number of hits in non secreted proteins; ^2^ p-value for enrichment in secretome; ^3^ number of domains in secretome; ^4^ number of domains in haustorial proteins; ^5^ tribes containing at least two instances of the domain with number of instances in parenthesis.
